# Gene-Diet Interaction between SIRT6 and Soybean Intake for Different Levels of Pulse Wave Velocity

**DOI:** 10.3390/ijms160714338

**Published:** 2015-06-24

**Authors:** Kexin Sun, Xiao Xiang, Na Li, Shaoping Huang, Xueying Qin, Yiqun Wu, Xun Tang, Pei Gao, Jing Li, Tao Wu, Dafang Chen, Yonghua Hu

**Affiliations:** 1 Department of Epidemiology and Biostatistics, Peking University Health Science Center, 38 Xueyuan Road, Beijing 100191, China; E-Mails: kexin_sun@bjmu.edu.cn (K.S.); doublex1990@live.cn (X.X.); xueyingqin@gmail.com (X.Q.); qywu118@163.com (Y.W.); tangxun@bjmu.edu.cn (X.T.); pei.gao@163.com (P.G.); jing2k9@gmail.com (J.L.); twu@bjmu.edu.cn (T.W.); dafangchen@bjmu.edu.cn (D.C.); 2 Fangshan District Center for Disease Control and Prevention, Beijing 102401, China; E-Mails: sophia8063@163.com (N.L.); fshcdcmb@yeah.net (S.H.)

**Keywords:** brachial ankle pulse wave velocity, atherosclerosis, genetic polymorphisms, inflammation, *ADIPOQ*, *SIRT6*, soybean, interaction

## Abstract

Soybean is a common food for the Chinese people. We aimed to investigate the risk for brachial ankle pulse wave velocity (baPWV) with inflammatory-related SNPs and soybean. baPWV was measured, and 16 inflammatory-related SNPs located on *ADIPOQ*, *CDH13*, *SIRT3*, *SIRT6*, *CXCL12*, *CXCR4*, *NOS1*, *PON1* and *CDKN2B* were genotyped in 1749 Chinese participants recruited from various communities. *ADIPOQ* rs12495941 (GT/TT *vs.* GG: crude OR = 1.27, *p* = 0.044) and *SIRT6* rs107251 (CT/TT *vs.* CC: crude OR = 0.74, *p* = 0.009) were associated with abnormal baPWV (baPWV ≥ 1700 cm/s). After adjustment for conventional environmental risk factors, rs12495941 was associated with abnormal baPWV (GT/TT *vs.* GG: adjusted OR = 1.43, *p* = 0.011), but the association between rs107251 and abnormal baPWV was not significant (CT/TT *vs.* CC: adjusted OR = 0.83, *p* = 0.173). The interaction between rs107251 and soybean intake for different levels of baPWV was statistically significant (*p* = 0.017). Compared with a high level of soybean intake, a low level of soybean intake can significantly decrease the risk of abnormal baPWV in individuals of rs107251 CT/TT genotypes (≤100 *vs.* >100 g/week: adjusted OR = 0.542, *p* = 0.003). In this study, associations between *ADIPOQ* rs12495941, *SIRT6* rs107251 and baPWV, as well as an interaction between *SIRT6* rs107251 and soybean intake for different levels of baPWV were found.

## 1. Introduction

Pulse wave velocity (PWV) is the speed at which the pulse wave propagates from heart to peripheral arteries [1]. It is widely used in clinical practice as a good surrogate marker of atherosclerosis [2]. A high pulse wave velocity indicates worsening arterial stiffness, which further implies a higher probability of atherosclerosis [3]. Atherosclerosis is known as a chronic inflammatory disease [4,5,6,7]. Genome-wide association studies (GWAS) focused on it have successfully identified numerous disease-associated SNPs located on inflammatory-related genes [8], such as 9p21 [9,10,11] and *CXCL12* [11]. The links between atherosclerosis and other inflammatory-related genes also have attracted much attention. Adiponectin is involved in regulating glucolipid metabolism and anti-inflammatory and antiatherogenic responses in the vascular system [12,13,14]. Cadherin 13 (*CDH13*) is an adiponectin receptor expressed in endothelial and smooth muscle cells [15,16]. The sirtuins (*SIRT*) are a family of highly-conserved nicotinamide adenine dinucleotide (NAD)^+^-dependent enzymes [17]. *SIRT3* and *SIRT6* have diverse biological functions in DNA repair processes, glucolipid metabolism and inflammation responses [18,19,20] and are suggested to have an impact on cardiovascular diseases (CVD), cancer and aging [21]. *CXCR4* is the receptor of *CXCL12*. The *CXCL12*/*CXCR4* axis plays an important role in atherogenesis [22]. The nitric oxide synthase 1 (*NOS1*) enzyme can affect the synthesis of NO (nitric oxide), which is involved in the pathogenesis of atherosclerosis [23]. Paraoxonase 1 (*PON1*) protects against atherosclerosis by preventing the oxidation of blood lipids [24]. These markers contribute to the initiation, propagation and activation of lesions in the arterial wall through different biologic pathways. In addition, atherosclerosis can also be affected by various environmental factors, such as exercise and diet [25,26]. Soybean is a common dish on Chinese dinner tables. Its nutritional value varies for different disease outcomes. It was reported that a soybean diet has a preventive effect on atherosclerosis [27]. However, whether soybean intake interacts with genetic polymorphisms for atherosclerosis was not clear.

Brachial ankle PWV (baPWV) calculates PWV using the pulse waves of brachial arteries and ankle arteries [28]. baPWV is significantly correlated with other surrogate markers of atherosclerosis, such as flow-mediated dilation (FMD) of brachial artery and carotid intima-media thickness (cIMT) [29]. In this study, we used baPWV as an independent predictor for atherosclerosis and chose 16 inflammatory-related SNPs located on *ADIPOQ*, *CDH13*, *SIRT3*, *SIRT6*, *CXCL12*, *CXCR4*, *NOS1*, *PON1* and *CDKN2B* using a candidate gene approach. The aims of this cross-sectional study were to: (1) identify those associated with baPWV among the chosen 16 candidate SNPs; (2) explore the association between soybean intake and baPWV; and (3) explore the interactions between soybean intake and selected SNPs in the first step for abnormally high baPWV.

## 2. Results

### 2.1. The Associations between Genetic Variations and baPWV

A total of 1749 Chinese Han participants were involved in the analysis. The average age was 58.3 ± 10.0 years old. Of the participants, 880 (50.3%) were male. Sixteen SNPs located on nine inflammation-related genes were genotyped. The associations between SNPs and baPWV were analyzed under additive, recessive and dominant models. The *ADIPOQ* rs12495941 polymorphism was significantly associated with baPWV under the dominant model. Compared with the GG genotype, the combined group of GT and TT genotypes (GT/TT) was positively associated with abnormal baPWV (≥1700 cm/s) (GT/TT *vs.* GG: OR = 1.27, *p* = 0.044). The *SIRT6* rs107251 polymorphism was significantly associated with abnormal baPWV under additive (per T allele: OR = 0.77, *p* = 0.004) and dominant models (CT/TT *vs.* CC: OR = 0.74, *p* = 0.009). These two SNPs were supposed to have influences on baPWV and were involved in the further analysis (Table 1).

**Table 1:** The associations between 16 inflammation-related SNPs and brachial ankle pulse wave velocity (baPWV).

SNP ID	Chr	Gene	Alleles	Reference Allele	MAF	HWE *p*-Value	*p*-Value		
							Additive	Dominant	Recessive
rs2228014	2	*CXCR4*	C/T	C	T (0.13)	0.691	0.591	0.879	0.151
rs182052	3	*ADIPOQ*	G/A	G	A (0.47)	0.788	0.335	0.595	0.286
rs12495941	3	*ADIPOQ*	G/T	G	T (0.38)	0.458	0.205	0.044	0.715
rs662	7	*PON1*	G/A	G	A (0.37)	0.884	0.677	0.622	0.898
rs3735590	7	*PON1*	C/T	C	T (0.14)	0.866	0.545	0.439	0.796
rs1333040	9	*CDKN2B*	T/C	T	C (0.29)	0.822	0.945	0.724	0.631
rs2383207	9	*CDKN2B*	G/A	G	A (0.33)	0.860	0.983	0.461	0.251
rs2383206	9	*CDKN2B*	A/G	A	G (0.47)	0.658	0.701	0.450	0.875
rs2297630	10	*CXCL12*	G/A	G	A (0.14)	0.676	0.896	0.880	0.356
rs1801157	10	*CXCL12*	G/A	G	A(0.23)	0.513	0.531	0.980	0.056
rs3825075	11	*SIRT3*	A/G	A	G (0.41)	0.777	0.483	0.849	0.290
rs2293050	12	*NOS1*	G/A	G	A (0.45)	0.587	0.086	0.258	0.089
rs6565105	16	*CDH13*	G/A	G	A (0.40)	0.911	0.269	0.298	0.466
rs1048612	16	*CDH13*	C/T	C	T (0.19)	0.617	0.688	0.563	0.727
rs3865188	16	*CDH13*	T/A	T	A (0.33)	0.785	0.539	0.646	0.565
rs107251	19	*SIRT6*	C/T	C	T (0.29)	0.965	0.004	0.009	0.052

Chr: chromosome; alleles: major allele/minor allele; MAF: minor allele (minor allele frequency); HWE: Hardy–Weinberger equilibrium; additive: *p*-values under additive models; dominant: *p*-values under dominant models; recessive: *p*-values under recessive models; the significance level for *p*-values was 0.05.

### 2.2. baPWV Depending on ADIPOQ rs12495941, SIRT6 rs107251 Polymorphisms and Different Levels of Soybean Intake

After adjustment for conventional environmental risk factors, as shown in Table 2, the *ADIPOQ* rs12495941 polymorphism was significantly associated with abnormal baPWV under additive (per T allele: OR = 1.23, *p* = 0.040) and dominant models (GT/TT *vs.* GG: OR = 1.43, *p* = 0.011). However, the association between the *SIRT6* rs107251 polymorphism and abnormal baPWV was not significant after adjustment for conventional environmental risk factors (per T allele: adjusted OR = 0.85, *p* = 0.128; CT/TT *vs.* CC: OR = 0.83, *p* = 0.173). Levels of soybean intake were not associated with baPWV after adjustment for conventional environmental risk factors (≤100 *vs.* >100 g/week: OR = 0.95, *p* = 0.629). The interaction between gender and soybean intake for different levels of baPWV was not significant (*p* = 0.768). (Table 2).

**Table 2:** Associations between and *ADIPOQ* rs12495941 and *SIRT6* rs107251 polymorphisms or levels of soybean intake baPWV.

Factors	baPWV *n* (%)		OR * (95% CI)	*p*
	≥1700 cm/s	<1700 cm/s		
***ADIPOQ* rs12495941 ^^^**				
GG	222 (33.7)	437 (66.3)	Ref	-
GT/TT	393 (36.1)	697 (63.9)	1.43 (1.09–1.88)	0.011
Per T Allele	-	-	1.23 (1.01–1.50)	0.040
***SIRT6* rs107251 ^#^**				
CC	344 (37.8)	566 (62.2)	Ref	-
CT/TT	271 (32.3)	568 (67.7)	0.83 (0.64–1.09)	0.173
Per T Allele	-	-	0.85 (0.68–1.05)	0.128
**Soybean Intake**				
>100 g/week	252 (35.2)	463 (64.8)	Ref	-
≤100 g/week	363 (35.1)	671 (64.9)	0.95 (0.77–1.17)	0.629

* Adjustment for age, gender, smoking status, drinking status, BMI, FBG, SBP, TG, HDL-C, vegetables and fruit intake, meat egg and milk intake, cereal intake and moderate-intensity exercise; ^^^ rs12495941: GG was the reference genotype, and G was the major allele. GT/TT represents the combined group of GT and TT genotypes and so does CT/TT; ^#^ rs107251: CC was the reference genotype, and C was the major allele; Ref: reference group.

### 2.3. Anthropometric, Lifestyle Characteristics and Biochemical Indices Depending on ADIPOQ rs12495941 and SIRT6 rs107251 Polymorphisms

There were no significant differences for the anthropometric, lifestyle or biochemical indices for different genotypes of *SIRT6* or *ADIPOQ* (Table 3).

**Table 3:** Anthropometric, lifestyle characteristics and biochemical indices of all participants depending on *SIRT6* rs107251 and *ADIPOQ* rs12495941 genotypes *.

Characteristics	*SIRT6* rs107251			*p*	*ADIPOQ* rs12495941			*p*
	CC	CT	TT		GG	GT	TT	
***n***	910	722	117	-	659	853	237	
**Age**	58.5 ± 9.8	57.9 ± 10.2	59.1 ± 10.6	0.292	58.6 ± 9.9	58.2 ± 10.0	57.7 ± 10.4	0.579
**Male, *n* (%)**	464 (51.0)	361 (50.0)	55 (47.0)	0.703	345 (52.4)	416 (48.8)	119 (50.2)	0.385
**Education, *n* (%)**				0.630				0.483
<9 years	299 (32.8)	259 (35.9)	45 (38.5)		239 (36.3)	281 (30.0)	83 (35.0)	
9–12 years	432 (47.5)	331 (45.8)	51 (43.6)		307 (46.6)	402 (47.1)	105 (44.3)	
>12 years	179 (19.7)	132 (18.3)	21 (17.9)		113 (17.1)	170 (19.9)	49 (20.7)	
**Smoke, *n* (%)**	402 (44.2)	341 (47.2)	53 (45.3)	0.461	317 (48.1)	367 (43.0)	112 (47.3)	0.146
**Drink, *n* (%)**	280 (30.8)	221 (30.6)	31 (26.5)	0.655	196 (29.7)	262 (30.7)	74 (31.2)	0.901
**Soybean Intake, *n* (%)**				0.505				0.594
>100 g/week	378 (41.5)	295 (40.9)	42 (35.9)		265 (40.2)	346 (40.6)	104 (43.9)	
≤100 g/week	532 (58.5)	427 (59.1)	75 (64.1)		394 (59.8)	507 (59.4)	133 (56.1)	
**Vegetables and Fruit Intake, *n* (%)**				0.569				0.937
<470 g/day	458 (50.3)	380 (52.6)	63 (53.8)		341 (51.7)	436 (51.1)	124 (52.3)	
≥470 g/day	452 (49.7)	342 (47.4)	54 (46.2)		318 (48.3)	417 (48.9)	113 (47.7)	
**Meat Egg and Milk Intake, *n* (%)**				0.231				0.218
<130 g/day	458 (50.3)	386 (53.5)	67 (57.3)		357 (54.2)	441 (51.7)	113 (47.7)	
≥130 g/day	452 (49.7)	336 (46.5)	50 (42.7)		302 (45.8)	412 (48.3)	124 (52.3)	
**Cereal Intake, *n* (%)**				0.945				0.485
<400 g/day	425 (46.7)	334 (46.3)	56 (47.9)		295 (44.8)	406 (47.6)	114 (48.1)	
≥400 g/day	485 (53.3)	388 (53.7)	61 (52.1)		364 (55.2)	447 (52.4)	123 (51.9)	
**Moderate-Intensity Exercise, *n* (%)**				0.347				0.067
<60 min/week	447 (49.1)	330 (45.7)	53 (45.3)		314 (47.6)	388 (45.5)	128 (54.0)	
≥60 min/week	463 (50.9)	392 (54.3)	64 (54.7)		345 (52.4)	465 (54.5)	109 (46.0)	
**BMI/kg/m^2^**	26.2 ± 3.8	26.1 ± 3.9	26.3 ± 4.1	0.931	26.0 ± 3.7	26.3 ± 4.0	26.1 ± 4.0	0.379
**FBG/mmol/L**	5.75 ± 2.23	5.82 ± 3.12	5.69 ± 2.47	0.827	5.9 ± 3.2	5.7 ± 2.3	5.7 ± 2.2	0.444
**SBP/mmHg**	141.7 ± 20.9	139.2 ± 21.4	139.9 ± 20.3	0.057	141.6 ± 26.0	140.7 ± 21.8	139.8 ± 20.7	0.524
**DBP/mmHg**	82.9 ± 16.1	81.7 ± 12.0	80.8 ± 10.1	0.052	81.9 ± 19.3	83.0 ± 16.4	83.6 ± 11.5	0.294
**TC/mol/L**	3.37 ± 0.94	3.30 ± 0.79	3.34 ± 0.98	0.275	3.32 ± 0.95	3.34 ± 0.83	3.36 ± 0.90	0.825
**TG/mmol/L**	1.81 ± 1.37	1.74 ± 1.47	1.80 ± 1.28	0.626	1.75 ± 1.56	1.78 ± 1.28	1.83 ± 1.41	0.783
**HDL-C/mmol/L**	1.14 ± 0.30	1.07 ± 0.35	1.01 ± 0.25	0.653	1.05 ± 0.28	1.06 ± 0.68	1.07 ± 0.30	0.540
**LDL-C/mmol/L**	2.68 ± 0.81	2.67 ± 0.78	2.64 ± 0.77	0.869	0.79 ± 0.03	0.81 ± 0.03	0.79 ± 0.05	0.440

* Continuous variables were described as the mean ± standard deviation. Categorical variables were described as frequency (proportion); BMI: body mass index; FBG: fasting blood glucose; SBP: systolic blood pressure; DBP: diastolic blood pressure; TC: total cholesterol; TG: total triglycerides; HDL-C: high-density lipoprotein cholesterol; LDL-C: low-density lipoprotein cholesterol.

### 2.4. Interactions between ADIPOQ rs12495941 and SIRT6 rs107251 Polymorphisms and Soybean Intake for Different Levels of baPWV

No interactions were found between *ADIPOQ* rs12495941 and soybean intake for different levels of baPWV in all participants (*p* = 0.800). However, a multiplicative interaction was found between *SIRT6* rs107251 and soybean intake in all participants (OR = 0.514, *p* = 0.017), which indicated that the effect of soybean intake on baPWV differed depending on the *SIRT6* rs107251 genotypes. For individuals of the rs107251 CT/TT genotypes, low soybean intake can significantly decrease the risk of abnormal baPWV (≤100 *vs.* >100 g/week: adjusted OR = 0.542, *p* = 0.003). (Table 4). After stratification by gender, the interaction between rs107251 and soybean intake continued to be significant in males (*p* = 0.033) but not in females (*p* = 0.122).

**Table 4:** Interactions between *ADIPOQ* rs12495941 and *SIRT6* rs107251 polymorphisms and soybean intake for different levels of baPWV.

Genotypes	Soybean Intake	baPWV *n* (%)		OR * (95% CI)	*p*	Interaction *p*-Value
		≥1700 cm/s	<1700 cm/s			
***ADIPOQ* rs12495941 ^^^**						
**GG**	>100 g/week	89 (33.6)	176 (66.4)	Ref	-	0.800
	≤100 g/week	133 (33.8)	261 (66.2)	0.96 (0.69–1.39)	0.232	
**GT/TT**	>100 g/week	163 (36.2)	287 (63.8)	1.37 (0.89–2.11)	0.159	
	≤100 g/week	230 (35.9)	410 (64.1)	1.12 (0.74–1.69)	0.596	
***SIRT6* rs107251 ^#^**						
**CC**	>100 g/week	137 (36.2)	241 (63.8)	Ref	-	0.017
	≤100 g/week	207 (38.9)	325 (61.1)	1.07 (0.74–1.56)	0.707	
**CT/TT**	>100 g/week	115 (34.1)	222 (65.9)	1.23 (0.81–1.88)	0.326	
	≤100 g/week	156 (31.1)	346 (68.9)	0.68 (0.46–1.01)	0.055	

* Adjustment for age, gender, smoking status, drinking status, BMI, FBG, SBP, TG, HDL-C, vegetables and fruit intake, meat egg and milk intake, cereal intake and moderate-intensity exercise; ^^^ rs12495941: GG was the reference genotype, and G was the major allele. GT/TT represents the combined group of GT and TT genotypes and so does CT/TT; ^#^ rs107251: CC was the reference genotype, and C was the major allele; the interaction between rs107251 and soybean: in males: OR = 0.431, *p* = 0.033; in females: OR = 0.528, *p* = 0.122; Ref: reference group.

## 3. Discussion

In the present candidate gene study, we evaluated the associations between 16 SNPs located on nine different inflammatory-related genes and baPWV. Among 16 SNPs, *ADIPOQ* rs12495941 and *SIRT6* rs107251 were associated with abnormal baPWV and were selected for the further analysis about the interactions. A multiplicative interaction was observed between *SIRT6* rs107251 and soybean intake, which indicated that the effect of soybean intake on baPWV differed depending on different genotypes of rs107251.

*ADIPOQ* rs12495941 is located in the intron 1 region, which was suggested to be associated with the risk factors for atherosclerosis, *i.e.*, adiponectin level [30], type 2 diabetes [31], body weight and waist and hip circumferences [31,32]. Since baPWV is a good surrogate marker of atherosclerosis, the association between rs12495941 and baPWV may partially be due to the relationship with these risk factors. The *SIRT6* gene is located on chromosome 19 (19p13.3). Full-length human *SIRT6* is a broadly-expressed, predominantly nuclear protein containing 355 amino acid residues [33]. *SIRT6* may influence the development of CVDs in several ways: (1) impact glycolipid homeostasis by controlling the expression of multiple related metabolic genes [21,34,35]; (2) Negatively regulate cardiac hypertrophy by attenuating insulin-like growth factor (IGF)-Akt signaling and, thus, impacting the development of heart failure [20]; (3) *SIRT6* is a critical regulator of vascular smooth muscle cell differentiation, which contributes to atherosclerosis [36]. rs107251 is an intron variant, which might be in linkage disequilibrium with the neighboring functional variant. TenNapel *et al.* [37] suggested that the CC or CT genotype at rs107251 displayed a more than five-year survival advantage compared to the TT genotype. Another study observed that T carriers of rs107251 had an increased risk for carotid plaque [38]. However, the current study suggested that rs107251 CC was the risk genotype for abnormal baPWV, and this association was not significant after adjustment for conventional risk factors. We suspect that this difference might be due to the existence of population heterogeneity. The first two studies were based on a Caucasian population and a multiethnic population (67% Caribbean Hispanic, 17% black and 15% white), respectively, while our study was based on a Chinese Han population. The genotype distributions of HapMap-CEU and HapMap-CHB populations are different (T allele frequency: 0.142 *vs.* 0.367). The genotype distributions of HapMap-JPT (T allele frequency: 0.322) were closer to those of the HapMap-CHB population, and a study based on a Japanese population reported that there were no association between *SIRT6* rs107251 T allele and diabetic nephropathy [39], which is a disease associated with atherosclerosis [40].

Though the biological functions of soy isoflavones, including prevention of osteoporosis [41], anti-photoaging effect [42] and anti-inflammatory activities [43], the association between soybean intake and different disease outcomes varied. The interactions between genetic polymorphisms and soybean intake were also reported. For example, high soy isoflavone intake may reduce the risk of breast cancer caused by *CYP1B1* risk genotypes [44]. Additionally, it was inversely associated with endometrial cancer among *SHBG* gene Asp/Asp carriers [45]. However, the *UGT1A1* rs2070959 GG genotype and low soy food intake may decrease the risk of endometrial cancer [46]. In addition, the studies concerning combined gene-soybean effects for atherosclerosis or its risk factors are limited. Wang *et al.* [47] suggested people with the CT haplotype of rs3846662 and rs3846663 and low soybean intake had a higher risk of being overweight and obesity. A high level of soybean intake can significantly reduce the levels of TC and LDL-C only for *BsmI* rs1544410 GG genotype carriers [48]. To our knowledge, this is the first study to investigate the role of the combined effect of *SIRT6* rs107251 and soybean for abnormal baPWV. Although the biological mechanism behind it is not well understood, the different effects of soybean intake on baPWV depending on different genotypes of rs107251 suggest that a soybean-rich diet might not be beneficial for everyone. After stratification by gender, significant interaction was observed only in male participants, which further implies that the role of soybean for males and females is different [49].

The strengths and potential limitation of the current study merit consideration. It is a relatively large study in a Chinese population with the measurement of inflammatory-related SNPs, the assessment of soybean consumption, other related dietary and lifestyle factors and baPWV. This allows us to explore not only the individual associations between the genetic or dietary risk factors and abnormal baPWV, but also the interactions of these risk factors for the surrogate marker of atherosclerosis. The study was carried out in a northern Chinese Han population, which greatly reduces the genetic heterogeneity. To improve data quality, the dietary and life style questionnaires were completed through the interviewer-administered method.

This study had some limitations. Firstly, given the fact that the study was a case-control design, results involving the dietary risk factors may be biased due to confounding; Secondly, although the food frequency questionnaire used in this study has been validated [50], measurement errors in food consumption are inevitable; Thirdly, total energy intake cannot be calculated in this study. However, logistic regressions involving soybean intake were all adjusted for other diet factors, which can partially reduce the influence on the results; Fourthly, the main effects of rs12495941 and rs107251 for baPWV were not validated in another independent dataset, so the results should be evaluated in larger epidemiology studies; Fifthly, factors like serum albumin and C-reactive protein may also affect baPWV, but were not assessed in this study; Finally, only 16 SNPs located on nine inflammation-related genes were selected based on literature reviews of their previously published associations with atherosclerosis and their potential biological functions. Thus, we cannot rule out the possibility that there might be other SNPs that interact with soybean intake and that can better explain the interactions observed in this study.

## 4. Experimental Section

### 4.1. Subjects

This community-based, cross-sectional study was conducted in Fangshan (a suburban district located in the southwest of Beijing, China). Three towns representing 3 different topographical areas (mountain, hill and plateau) within the district were selected for recruitment, for the socio-economic levels and medical conditions differ among these areas. The inclusion criteria for participants were as follows: (1) age ≥40 years old; (2) capable of cooperating during physical examinations. The exclusion criteria were as follow: (1) ethnic group was not Han; (2) not a permanent resident. 2049 participants completed baPWV measurement and diet investigation, among which 256 refused to give blood samples, 44 were excluded for the differences between their left side and right side baPWVs being more than 500 cm/s, which might be an indicator for artery occlusion. At last 1749 subjects were included in the analysis of this study.

### 4.2. baPWV Measurement

baPWV was measured by trained medical staff using automatic devices (BP-203PRE-III, Colin, Japan). After a participant lied down for 3 min, 4 blood pressure cuffs were bound to the upper limbs and ankles. The pulse waves of four limbs were automatically recorded using oscillography. PWV was calculated by dividing the distance between two measuring points by the time difference of pulse waves of two points. This method was proven to have high reliability and validity [51]. In this study, the mean of left side and right side baPWVs was taken as one’s final baPWV. The binary baPWV outcome was generated by using the cutoff point of 1700 cm/s for predicting cardiovascular events suggested by Tomiyama *et al.* [52], *i.e.*, baPWV ≥ 1700 cm/s was defined as abnormal baPWV in this study.

### 4.3. Measurement of Soybean Consumption and Other Dietary Factors

The food intake questionnaire used in this study was a shortened version of the FFQ from Shanghai Women’s Health Study (SWHS). The validity and reproducibility of the FFQ was reported elsewhere [50]. The food list was revised based on the dietary habits of Beijing rural residents, including the items of cereal foods, vegetables, fruit, egg, milk and soybean. During the face-to-face surveys, subjects were first asked at which frequency level they consumed each food (feasible choices including daily, weekly, monthly, yearly or never). Questions of how many times at each level they consumed these foods and the average amount of consumption per time followed. A standard portion size measured in liang (1 liang = 50 g), a weight unit commonly used in China, was specified for each food (e.g., bowl, glass, serving). According to the recorded frequency and amount of food intake per time, the average weekly intake amount for soybean and average daily intake amount for other dietary factors were calculated. Diet factors were dichotomized by their median intake amount.

### 4.4. Anthropometric and Biochemical Indices Determinations

Demographic information was collected by a standard questionnaire. Surveys were carried out by trained and qualified staff. The definition of being a current smoker was having at least 1 cigarette per day and lasting for at least 6 months. The definition of a current drinker was having at least 50 milliliters of white spirits per week and lasting for at least 6 months. The weekly frequency and duration of moderate-intensity exercise were also asked.

Anthropometric variables, such as weight and height, were measured by trained doctors and nurses. After being calmed down for 5 min, sitting blood pressure was examined 3 times by brachial blood pressure meters (HEM-7200, Omron Healthcare, Kyoto, Japan). The mean of the second and the third observed values was taken as one’s final blood pressure. BMI was calculated as weight/height^2^ (kg/m^2^).

Participants were asked to fast at least for 8 h before the collection of blood samples. Fasting venous blood was drawn for genotyping and biochemical indices measurements. All blood samples were tested at Peking University Health Science Center Key Laboratory of Epidemiology for fasting blood glucose (FBG), total cholesterol (TC), total triglycerides (TG), high-density lipoprotein cholesterol (HDL-C) and low-density lipoprotein cholesterol (LDL-C).

### 4.5. Genotyping of SNPs

DNA was extracted from peripheral blood leucocytes. Inflammatory-related SNPs were selected using the literature review method. Genotyping of rs182052, rs12495941, rs6565105, rs1048612, rs3865188, rs3825075, rs107251, rs2293050, rs2297630, rs1801157, rs2228014, rs662, rs3735590, rs1333040, rs2383207 and rs2383206 was performed using time-of-flight mass spectrometry genotyping technology with a MassARRAY^®^ genetic analysis system (Sequenom Inc., San Diego, CA, USA) following the manufacturer’s protocol. Genotypes were assessed by MassARRAY^®^ Typer Analyzer Version 4.0. The call rates for 16 SNPs were all above 99.5%.

### 4.6. Statistical Analysis

The distributions of SNPs were analyzed for deviation from Hardy–Weinberg equilibrium (HWE) using Pearson’s χ^2^ test, and no violation was found for any SNP. HWE *p*-values for all SNPs are shown in Table 1. Dominant, recessive and additive genetic models were assumed for every SNP. For a polymorphism with two alleles (A and a, A was the risk allele), three genotypes (AA, Aa, aa) are to be tested for association. AA and Aa genotypes are pooled together in the dominant model, while Aa and aa genotypes are pooled together in the recessive model. The additive model indicates a linear relation between the number of risk allele and disease risk [53].

Continuous variables were described as the mean ± standard deviation and the Student *t*-test was adopted to compare means across groups. Categorical variables were described as frequency and proportion, and Pearson’s χ^2^ test was used to compare frequencies between groups.

Unconditional logistic regression (ULR) was performed to estimate the associations between baPWV and SNPs. All ORs were adjusted for variables proven to be associated with baPWV or atherosclerosis previously, including age, gender, smoking status, drinking status, BMI, FBG, systolic blood pressure (SBP), TG and HDL-C. To estimate the interaction effect, a multiplicative term of two variables was included in the regression model. The data were analyzed using SPSS Version 16.0 for windows (SPSS Inc., Chicago, IL, USA).

Statistical power for the interactions between rs107251, rs12495941 and soybean intake was calculated by QUANTO 1.2.4 (Available online: http://hydra.usc.edu/gxe). The statistical power based on the current sample size was over 80%.

### 4.7. Ethics Statement

The study design was explained to every subject during recruitment. Every participant gave written informed consent. This project was approved by the Ethics Committee of Peking University Health Science Center, Beijing, China.

## 5. Conclusions

Among 16 inflammatory-related SNPs located on *ADIPOQ*, *CDH13*, *SIRT3*, *SIRT6*, *NOS1*, *CXCL12*, *CXCR4*, *PON1* and *CDKN2B*, *ADIPOQ* rs12495941 and *SIRT6* rs107251 polymorphisms were associated with baPWV. An interaction between *SIRT6* rs107251 and soybean intake for abnormally high baPWV was found. A low level of soybean intake can significantly decrease the risk of abnormal baPWV in individuals of rs107251 CT/TT genotypes, but not in those of the CC genotype. The results should be evaluated in larger epidemiology studies.
